# CXCL17-derived CD11b^+^Gr-1^+^ myeloid-derived suppressor cells contribute to lung metastasis of breast cancer through platelet-derived growth factor-BB

**DOI:** 10.1186/s13058-019-1114-3

**Published:** 2019-02-12

**Authors:** Ya-Ling Hsu, Meng-Chi Yen, Wei-An Chang, Pei-Hsun Tsai, Yi-Chung Pan, Ssu-Hui Liao, Po-Lin Kuo

**Affiliations:** 1 Graduate Institute of Medicine, College of Medicine, Kaohsiung Medical University, Kaohsiung, 807 Taiwan; 2 Department of Medical Research, Kaohsiung Medical University Hospital, Kaohsiung, 807 Taiwan; 3 Center for Biomarkers and Biotech Drugs, Kaohsiung Medical University, Kaohsiung, 807 Taiwan; 4 Graduate Institute of Clinical Medicine, College of Medicine, Kaohsiung Medical University, No. 100, Shih-Chuan 1st Road, Kaohsiung, 807 Taiwan; 5 Department of Emergency Medicine, Kaohsiung Medical University Hospital, Kaohsiung, 807 Taiwan; 6 Division of Pulmonary and Critical Care Medicine, Kaohsiung Medical University Hospital, Kaohsiung, 807 Taiwan; 7 Institute of Medical Science and Technology, National Sun Yat-Sen University, Kaohsiung, 804 Taiwan

**Keywords:** Breast cancer, CXCL17, Lung metastasis, Myeloid-derived suppressor cells, PDGF-BB

## Abstract

**Background:**

Metastasis is the major cause of death from breast cancer. Colonization and adaption of metastatic cells in distant organs is a rate-limiting step of the cancer spreading. The underlying mechanisms responsible for the colonization of breast cancer to lung metastatic niches are not fully understood.

**Methods:**

Specific gene contributions to lung metastasis were identified by comparing gene profiles of 4T1 tumors metastasizing to various organs via microarray. The oncogenic properties CXCL17 were examined by in vivo spontaneous metastasis mouse model. The chemotactic activity of CXCL17 on CD11b^+^Gr-1^+^ myeloid-derived suppressor cells (MDSCs) was examined by both in vitro and in vivo models. The therapeutic effects of MDSC depletion and platelet-derived growth factor-BB (PDGF-BB) inhibition were examined by orthotic models.

**Results:**

Here, we demonstrate that breast cancer cells secrete CXCL17, which increases the accumulation of CD11b^+^Gr-1^+^ MDSCs in the lungs. Metastatic lung-infiltrating CD11b^+^Gr-1^+^ MDSCs induce angiogenesis in the lungs and facilitate cancer extravasation and survival that ultimately promote lung metastases. CXCL17 increases CD11b^+^Gr-1^+^ MDSCs to express PDGF-BB, which not only contributes to CD11b^+^Gr-1^+^ MDSC-mediated angiogenesis in the lung metastatic niche, but is also involved in the colonization of breast cancer. Consequently, both CD11b^+^Gr-1^+^ MDSC depletion and PDGF receptor inhibitor effectively prevents CXCL17-driven lung metastasis in breast cancer. More importantly, patients with high levels of CXCL17 have shorter distant metastasis-free and overall survival rates, indicators of poor prognosis.

**Conclusion:**

Our study reveals that MDSCs derived by CXCL17 contribute to the establishment of a lung metastatic niche by PDGF-BB secretion and provide a rationale for development of CXCL17 or PDGF-BB antagonists to inhibit or prevent lung metastasis in cases of breast cancer.

**Electronic supplementary material:**

The online version of this article (10.1186/s13058-019-1114-3) contains supplementary material, which is available to authorized users.

## Introduction

Breast cancer is the most common malignant disease affecting women worldwide. Recent statistics indicate that breast cancer is estimated to account for 30% of all new cancer diagnoses in women [1]. Despite recent advances in breast cancer diagnosis and therapeutic techniques such as hormonal and target therapy, chemotherapy, and radiotherapy, patients under remission may still experience breast cancer relapse and spread [2, 3]. Therefore, improving our understanding of the biology of cancer metastasis may lead to the discovery of more effective strategies for improving the prognosis and treatment of advanced breast cancer.

Metastasis is the major cause of almost all breast cancer deaths. Secondary growth in breast cancer primarily occurs in the lymph nodes, bones, liver, lungs, and brain [4]. A remarkable feature of this multi-step and highly-organized cell biological process is the variation in metastatic organ-specific tropism depending on the tumor type [5, 6]. One of the reasons for organ-specific tropism of cancer cells is the formation of a permissive microenvironment, known as the metastatic niche, in target organs. Myeloid-derived suppressor cells (MDSCs) have been proven to play a prominent role in the establishment of the metastatic microenvironments [7, 8]. MDSCs are a heterogeneous population of immune cells that are myeloid origin precursors and relatively immature. Currently, MDSCs are divided into two distinct subsets, polymorphonuclear (PMN)- or granulocytic (G)-MDSC, and monocytic (M)-MDSC [9]. G-MDSCs (CD11b^+^Ly6C^int/lo^Ly6G^+^) share many phenotypic and functional features of neutrophils, whereas M-MDSCs (CD111b^+^Ly6C^+^Ly6G^−^) are related to monocytes [10, 11]. The granulocytic nature of CD11b^+^Gr-1^+^ cells has been detected in the lung tissue of mice with mammary adenocarcinoma [12]. Growing evidence supports the hypothesis that MDSCs exert their pro-tumorigenic effects by suppressing T and B cell functions and promoting tumor angiogenesis, proliferation, survival, and metastasis [13]. However, it remains unclear why and how MDSCs accumulate in the lungs, creating a permissive microenvironment for metastasizing breast cancer cells.

Chemokines, a superfamily of small chemotactic cytokines with the ability to bind to G-protein-coupled receptors, play a critical role in the recruitment of various immune cells to specific tissues, in a variety of physiological and pathological conditions [14, 15]. Chemokine (C-X-C motif) ligand 17 (CXCL17) is a novel 119 amino acid CXC chemokine, which has been reported to express in colon and breast cancers and promotes cancer progression [16–18]. CXCL17 has been identified as an independent prognostic factor for overall survival and progression-free survival for patients with hepatocellular carcinoma, since it is negatively correlated with CD4^+^ T cell accumulation, but positively regulates CD68^+^ macrophage infiltration [19]. In addition, ectopic expression of CXCL17 increases tumorigenesis and cancer growth by recruitment of CD11b^+^Gr-1^high^F4/80^−^ cells in the primary site of colon cancer [18]. Herein, we describe the pathogenic role of CXCL17 in the formation of a lung metastatic niche in the case of breast cancer. In addition, CXCL17 levels have been correlated with breast cancer metastases among patients with breast cancer. Therefore, we propose that the evaluated expression of CXCL17 in breast cells might play a pivotal role in the MDSC-driven lung shaping by platelet-derived growth factor-BB (PDGF-BB) that contributes to lung metastasis.

## Materials and methods

### Cell lines and reagents

Human breast cancer MDA-MB-231, murine breast cancer 4T1**,** and endothelial C166 cell lines were purchased from American Type Culture Collection (ATCC, Manassas, VA). MDA-MB-231-RFP-Luciferase cells were obtained from GenTarget Inc. All cell lines were assessed for mycoplasma contamination using MycoAlert Mycoplasma Detection Kit (Lonza, Walkersville, MD) every 6 months. 4T1 cells were maintained in RPMI1640 supplemented with 10% fetal bovine serum (FBS) and antibiotics. MDA-MB-231 and MDA-MB-231-RFP-Luc cells were cultured in Leibovitz’s L-15 medium supplemented with 10% FBS (Life Technologies, Grand Island, NY). C166 cells were cultured in with DMEM medium supplemented with 10% FBS. Recombinant mouse (rm) and human (rh) CXCL17 was obtained from R&D Systems (Minneapolis, MN, USA). CID 2745687 and DMPQ dihydrocloride (5,7-Dimethoxy-3-(4-pyridinyl)quinoline dihydrochloride, DMPQ 2HCl) were obtained from Torcis (Minneapolis, MN, USA). PDGF-BB was obtained from ProSpec (Ness Ziona, Israel).

### Mouse studies

All mice procedures were conducted and approved in accordance with the Institutional Animal Care and Use Committee at Kaohsiung Medical University. BALB/c mice (female, 5-week-old) were treated with rmCXCL17 for 14 days (1 μg/mouse, 2 times/week, *n* = 6 per group) by intra-tracheal route, and 4T1 cells (500,000 cells per fat pad) were implanted into the mammary fat pads and allowed to spontaneously metastasize to the lung for 24 days. For lung metastasis of a human breast cancer model, athymic nude mice (6-week-old females, *n* = 6 per group) were treated with rmCXCL17 protein (1 μg/mouse, 2 times/week) for 14 days, then human MDA-MB-231 or MDA-MB-231-RFP-Luc cells were injected via the tail vein for the indicated times (90 days for lung metastasis and 48 h for extravasation). Tumors in primary sites and the lungs were harvested after the indicated times, fixed and embedded in paraffin, sectioned, and immunostained with antibodies against CD31 antibodies (Catalog #ab28364, dilution 1:100). All lung nodules in both lung lobes were calculated and tumor nodules occurring in the same site of sequential sections were only calculated once. Quantitative studies of stained sections were performed independently by three researchers in blinded fashion. The total number of tumor nodules per whole lung lobe was counted and averaged among the animals of each group. Quantitative analyses of CD31 staining were performed with the image analysis program ImageJ. Assays were carried out three times, and three random fields per sample were analyzed in five high-power fields (100 magnification [10 objective and 10 ocular]). For L4T1 generation, 4T1 cells were transplanted into BALB/c mice from mammary fat pads. The animals were then sacrificed on day 24, and the 4T1 cells in the lungs were isolated by mincing, collagenase type I digestion, and filtering. 4T1 cells were cultured in a growth medium containing 10% fetal bovine serum and 1% penicillin–streptomycin and expanded for second-round transplantation. The 4T1 sub-line of transplantation was designated as L4T1. For MDSC depletion studies, mice were treated with isotype or anti-Gr-1 antibody (Bio X Cell, West Lebanon, NH) at 0.25 mg/mouse intraperitoneal injections every 4th day, and 4T1 cells were injected via tail vein into the mice (six per group). The control group received intraperitoneal injections of purified rat immunoglobulins. For PDGF receptor inhibition studies, mice were treated with imatinib mesylate (Sigma-Aldrich) (100 mg/kg) by oral gavage three times weekly, and 4T1 cells were transplanted into mice from mammary fat pads (*n* = 6 per group). The control group received oral gavage of vehicle control (10% ethanol and 90% corn oil).

### Isolation of CD11b^+^F4/80^+^, CD11b^+^Gr-1^+^, and CD11b^+^Gr-1^−^ cells from lungs of mice

Murine lung tissue was dispersed using 2% collagenase A and 0.75% DNase I (Roche Diagnostics GmbH, Germany) in RPMI1640 medium supplemented with 10% FBS at 20 °C for 1 h. CD11b^+^ F4/80^+^ cells were isolated from the lungs of mice by magnetic cell sorting using mouse CD11b and F4/80 antibodies conjugated with magnetic beads. CD11b^+^Gr-1^+^ and CD11b^+^Gr-1^−^ cells were isolated using MDSC Isolation Kit (MACS, Miltenyi Biotec) according to the manufacturer’s instructions.

### Microarray

Microarray experiment procedures were carried out following the manufacturer’s protocols. Total RNA was amplified by an Agilent Quick Amp Labeling Kit (Agilent Technologies, USA). Cy-labeled cRNA (0.3 μg) was cleaved to an average size of about 50–100 nucleotides by incubation with fragmentation buffer (Agilent Technologies) at 60 °C for 30 min. Equal Cy-labeled cRNA was pooled and hybridized to Agilent SurePrint G3 Mouse GE 8x60K Microarray (Agilent Technologies, USA), then scanned by an Agilent microarray scanner (Agilent) at 535 nm for Cy3 and 625 nm for Cy5. Scanned images were analyzed by Feature Extraction software 10.5 (Agilent Technologies), and image analysis and normalization software was used to quantify signal and background intensity for each feature, which substantially normalized the data using the rank-consistency-filtering LOWESS method.

### Measurement of secreted factors

The levels of angiogenic factors were determined by Luminex Assays (R&D Systems). CXCL17 levels were assessed by human or mouse CXCL17 ELISA kits (Cusabio Biotech, Wuhan, China).

### Tube formation assay

C166 (4 × 10^5^ cells/well) were then plated on reduced growth factor Matrigel (200 μL/well) (BD Biosciences, San Josè, CA) in 48-well plates and treated with conditioned medium (CM) (50%) of CD11b^+^Gr-1^+^ MDSCs isolated from normal mice for 3 h. Photographs were taken using a Nikon fluorescence microscope. The total tube area was quantified as mean pixel density obtained from image analysis of three random microscopic fields using ImageJ software.

### Migration, transendothelial migration, and colony formation

Cell migration assays were conducted using the 3- or 8-μm inserts (EMD Millipore). PKH26-labeled CD11b^+^Gr-1^+^ or 4T1 cells (1 × 10^5^ cells/well) were seeded onto inserts, and CXCL17 (1 or 10 ng/ml in RPMI1640 medium containing 1% FBS) or CMs of CD11b^+^Gr-1^+^ MDSCs were added to the bottom wells for 24 h as chemoattractant. Migratory cells were counted using a fluorescence microscope. For transendothelial migration, C166 were seeded onto inserts with polyester membranes of 3 (for MDSCs) or 8 μm (for 4T1 cells) pore size (EMD Millipore) and cultured for 2 days to allow a confluent monolayer to form. PKH26-labled CD11b^+^Gr-1^+^ MDSCs or 4T1 cells were seeded onto C166-coated inserts for 24 h. CMs of CD11b^+^Gr-1^+^ MDSCs isolated from normal or 4T1-bearing mice were placed at the bottom wells to be acted as chemoattractant, and the migratory cells were made visible using a fluorescence microscope. For colony formation, 4T1 cells (1 × 10^3^) were seeded into a 60-mm dish and then treated with CM of CD11b^+^Gr-1^+^ MDSCs isolated from normal or 4T1-bearing mice (50%). After 7 days, the colonies were fixed by formalin (1%), then stained by crystal violet.

### CXCL17 expression and Kaplan-Meier analyses of breast cancer patients

The SurvExpress contain 189 (Sotiriou Van de Vijver Breast GSE2990) and 327 (Kao Hung Breasr GSE20685) breast cancer patients with follow-up time intervals [20]. The data were used to estimate the prognostic significance of the *CXCL17* transcript for metastasis curve, while Kaplan-Meier plotter database was used to evaluate distant metastasis-free survival [21].

### Statistical analyses

Data were expressed as mean ± standard deviation (SD) or standard error of mean (SEM). Two treatment groups were compared by Student’s *t* test. Multiple group comparisons were performed by two-way analysis of variance with Tukey’s post hoc test. Metastatic quantifications were assessed with a Mann-Whitney *U* test. GraphPad Prism version 7.04 (GraphPad Inc., La Jolla, CA) was used for statistical analyses. Results were considered statistically significant when *P* < 0.05.

## Results

### The upregulation of CXCL17 in lung metastatic breast cancer

**Fig. 1:**
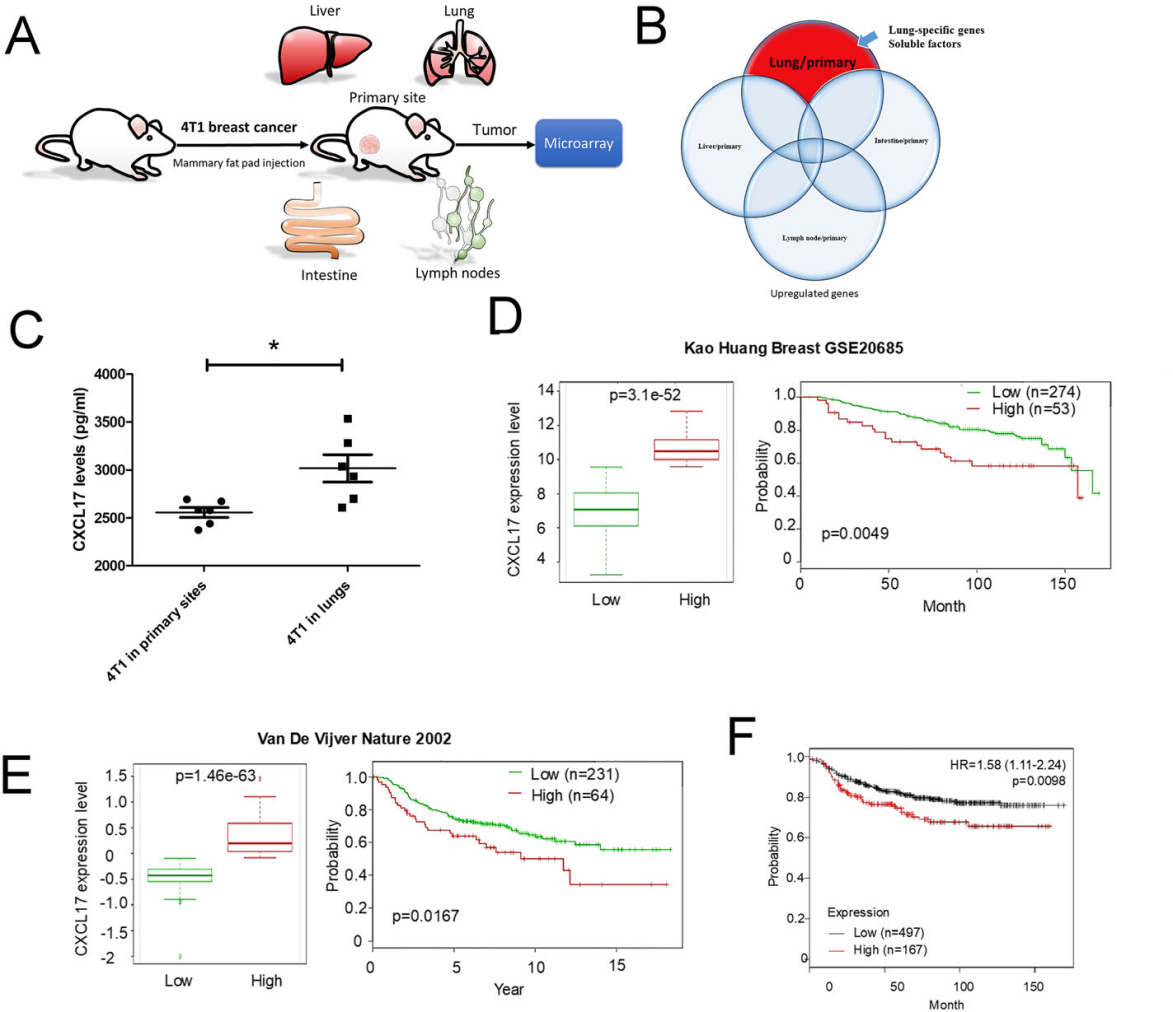
CXCL17 is upregulated in lung metastatic breast cancer cells. **a** Scheme of the animal model. 4T1 cells (500,000 cells per fat pad) were implanted into the mammary fat pads and allowed to spontaneously metastasize to the lung for 24 days. Whole primary tumor in mammary or tumor nodules in the lungs (*n* = 3), livers (*n* = 4), and intestine (*n* = 4) and lymph nodes (tumor nodules = 7) were subjected to gene profiling. **b** Specific gene profile of breast cancer metastasized to lung. **c** The upregulation of CXCL17 protein in lung metastatic 4T1 cells. 4T1 cells were implanted into mice in an orthotic model. The expression of CXCL17 protein of 4T1 cells isolated from primary sites (mammary) or lung tissue (six pairs of 4T1 cell isolated from in mammary and lung) was assessed by ELISA after 48-h incubation. **d** In the left panel, the box plot generated from Kao Huang Breast GSE20685 dataset of SurvExpress showed the amount of CXCL17 expression in each group. The right panel showed the Kaplan-Meier time to metastasis curve. **e** The results were from Van De Vijver Nature 2002 dataset of SurvExpress. The left and right panels showed CXCL17 expression and the Kaplan-Meier time to metastasis curve respectively. The group was divided according to the “Maximize Risk Groups” in SurvExpress website. **f** Kaplan-Meier distant metastasis-free survival via KM plotter database. High and low CXCL17 expression groups were divided according to “Auto select best cutoff” in the KM plotter website. Each value is the mean ± SD of three determinations; *p < 0.05

To identify specific candidate secretory factors that mediate breast cancer metastasis by shaping lung niches, we found upregulated genes only in lung metastatic 4T1 cells, but not in lymph, intestinal, and liver 4T1 cells (Fig. 1a, b). Thirty-three secretory factors were found in lung metastatic 4T1cells (Table 1). Among these genes, we focused on CXCL17, which is upregulated at the highest fold (24.092-fold) of lung metastatic 4T1 cells, compared to the primary 4T1 in the fat pads of mice. The ELISA also confirmed that CXCL17 was enhanced at protein level in lung metastatic 4T1 (L4T1) cells, compared to 4T1 cells in tumors of primary sites (Fig. 1c). We also assessed the association between CXCL17 expression and metastasis in breast cancer via the online databases SurvExpress and KM plotter (PMID: 24066126 and PMID: 20020197). In two independent datasets from SurvExpress (PMID: 21501481 and PMID: 11823860), distant metastasis-free survival revealed that the high-risk group expressed high levels of CXCL17 and had shorter time to metastasis in both datasets (Fig. 1d, e). Similar results were shown in Kaplan-Meier survival analysis (Fig. 1f). The results suggest that CXCL17 might be associated with metastasis in breast cancer.

**Table 1:** Upregulation secretory factors in 4T1 tumors in the lungs of mice

Gene	Folds	Gene	Folds	Gene	Folds	Gene	Folds
CCL1	2.468	CXCL15	38.937	FGF4	19.932	HGF	8.082
CCL4	2.028	CXCL17	24.092	FGF10	9.988	VEGF	7.924
CCL5	4.030	CXCL12	9.338	FGF1	7.007	ET-1	6.417
IL33	3.619	CXCL10	4.05	FGF18	4.833	IGF1	2.990
IL12	3.561	CXCL9	3.279	GDF5	9.746	TGFb2	3.768
IL17r	2.283	MMP7	3.222	GDF15	7.745	LTF	10.370
WNT4	2.696	MMP8	8.501	TNF	3.612	PDGFB	2.401
WNT6	8.163	MMP15	3.519	TNFSF10	3.424		
WNT10A	3.788			TNFSF13B	3.102		

4T1 cells were implanted into the mammary fat pads and allowed to spontaneously spread to the lung for 24 days. Whole primary tumor in mammary and tumor nodules in lungs (tumor nodules = 8) were subjected to microarray

### CXCL17 mediates lung metastasis of breast cancer in vivo

**Fig. 2:**
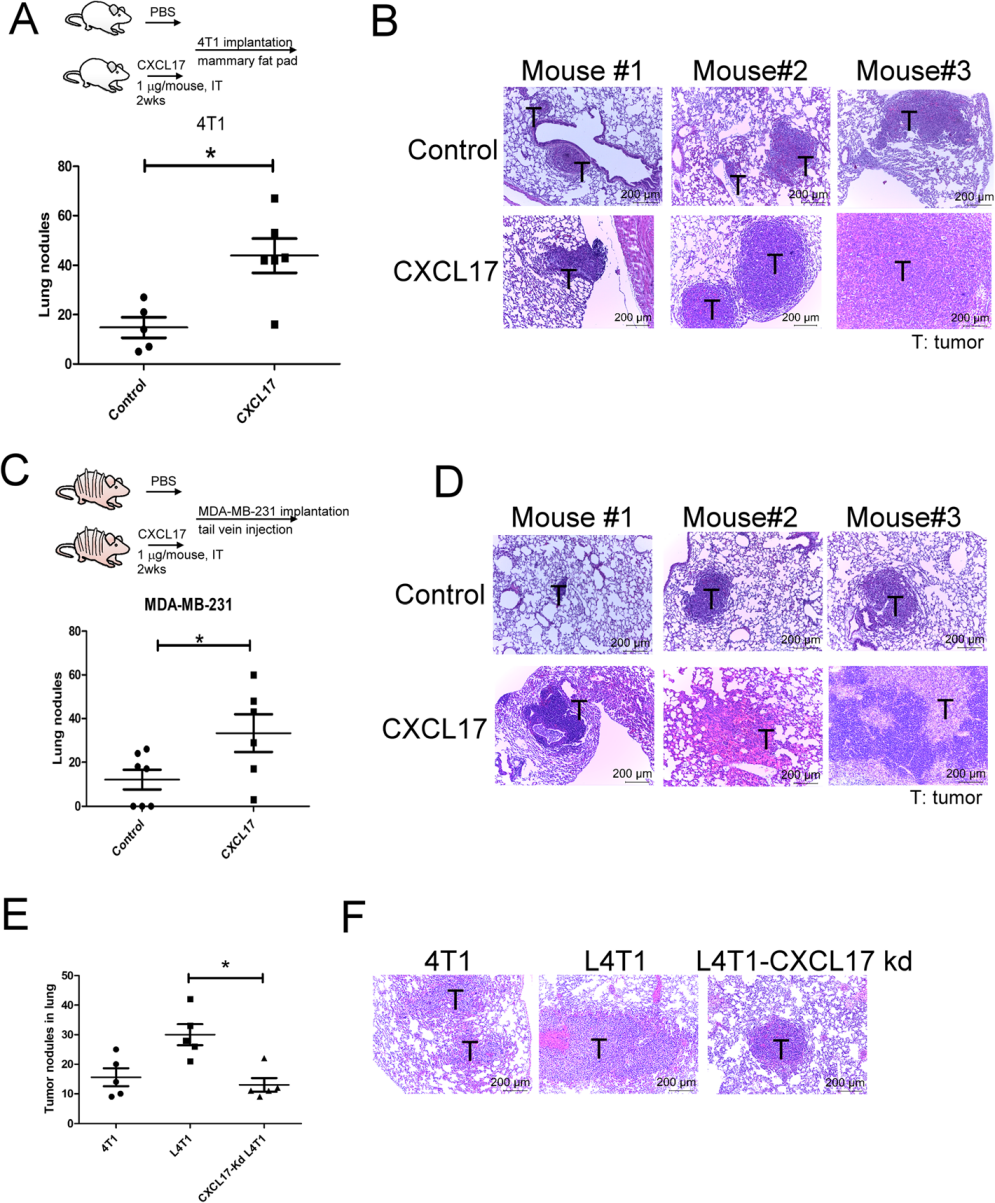
CXCL17 increased lung metastasis in vivo. **a** CXCL17 increased the lung metastasis of 4T1 cells in an orthotropic model. The total number of tumor nodule per whole lung lobes was counted and averaged among the animals of each group. **b** The H&E staining of tumor sections. BALB/c mice were treated with PBS or recombinant mouse CXCL17 protein by intra-tracheal administration for 14 days (1 μg/mouse, 2 times/week, *n* = 6 per group). 4T1 were implanted into the fat pads of mice. Tumor nodules of 4T1 in the primary site and lungs were collected after 24 days of injections. **c** CXCL17 increased lung metastasis in human breast MDA-MB-231. Nude mice were treated with PBS or recombinant CXCL17 protein by intra-tracheal administration for 14 days (1 μg/mouse, 2 times/week, *n* = 6 per group). MDA-MB-231 cells were implanted into mice by tail vein injection. The MDA-MB-231 tumor nodules in the lungs of mice were collected after 90 days of injections. **d** The H&E staining of tumor sections. **e** Knockdown of CXCL17 decreased the spontaneous metastatic ability of L4T1 cells. **f** The H&E staining of tumor sections. CXCL17-knockdown-L4T1 were implanted into the fat pads of mice (*n* = 6 per group). Tumor nodules in the lungs were collected after 24 days of injections. Representative lung tissue sections were stained with H&E and photographed at × 100 magnification. Each value is the mean ± SEM; Mann-Whitney *U* test was performed, *p < 0.05. T tumor

To assess whether CXCL17 promotes lung metastasis through metastatic niche establishment, we treated mice with rm (recombinant mouse protein) CXCL17 via intra-tracheal administration and then implanted breast cancer cells by orthotropic graft or tail vein injection. Compared to control mice, rmCXCL17 treatment increased the numbers of tumor nodules in the lungs of mice that had received tumor implantation in an orthotropic model (Fig. 2a, b). In a second approach, we implanted human breast MAD-MB-231 cells into mice by tail vein injection after rmCXCL17 treatment for 2 weeks. Compared to control mice, more and larger tumor nodules were observed in the lungs of mice pre-treated with rmCXCL17 (Fig. 2c, d). Histological analysis revealed that mice treated with rmCXCL17 had a higher number of metastatic foci, and with larger sizes, compared with mice injected with PBS (Fig. 2d). Moreover, knockdown of CXCL17 decreased the spontaneous metastatic ability of L4T1 cells (Fig. 2e, f). However, CXCL17 did not change cell proliferation, colony formation, and migration of 4T1 breast cancer cells in vitro (Additional file 1: Figure S1A to S1C). These data indicate that CXCL17 might contribute to the supportive metastatic niche formation rather than cancer cell growth or mobility regulation, for breast cancer spreading.

### CXCL17 is responsible for the formation of lung metastatic niche by recruiting PDGF-BB-expressing MDSCs

**Fig. 3:**
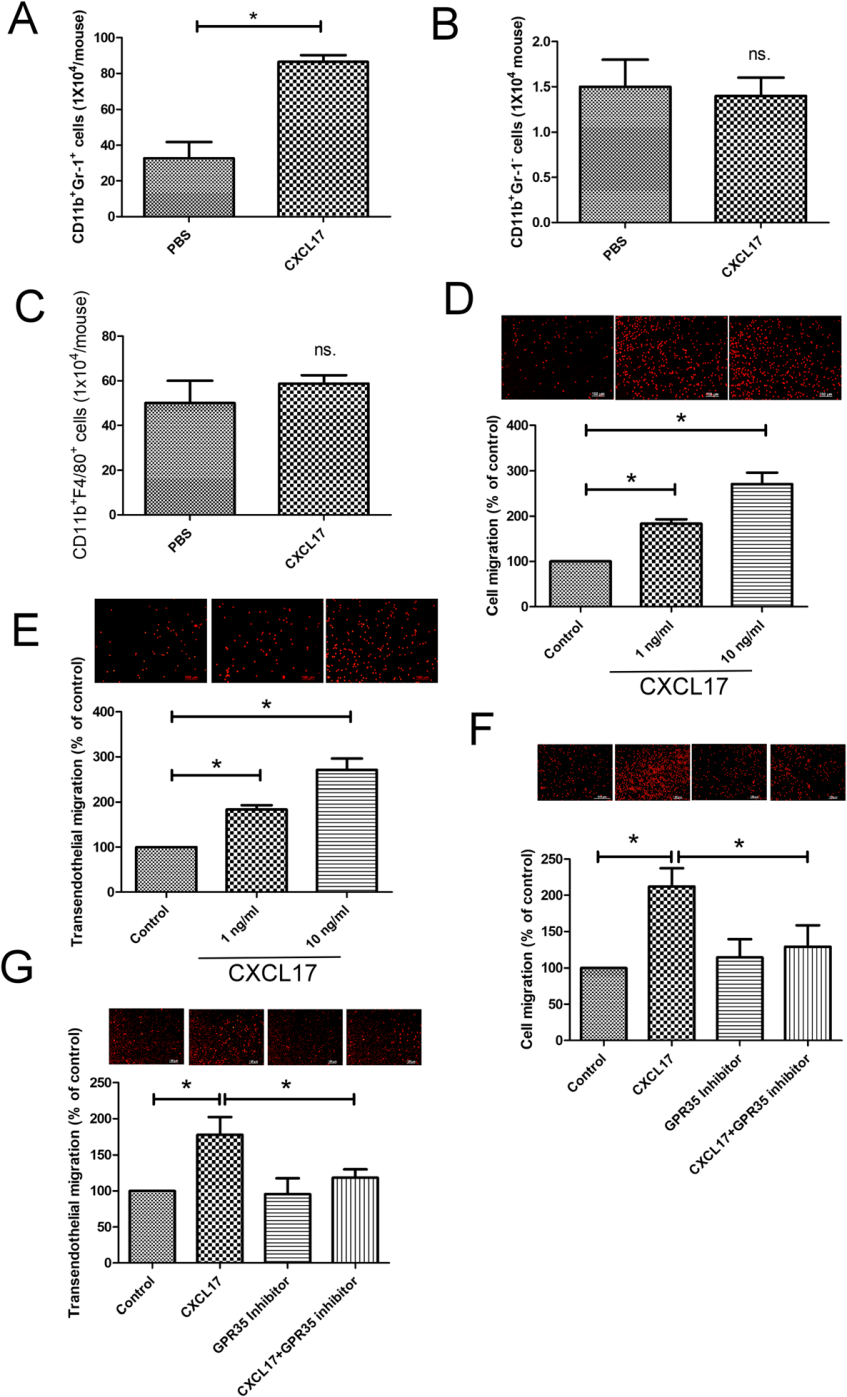
CXCL17 increases the recruitment of MDSCs in metastatic lungs of mice. The effect of CXCL17 in the recruitment of CD11b^+^Gr-1^+^ MDSCs (**a**), CD11b^+^Gr-1^−^MDSCs (**b**), and CD11b^+^F4/80^+^ macrophages (**c**) in the lungs of mice. BALB/c mice were treated with PBS or recombinant mouse CXCL17 protein by intra-tracheal administration for 14 days (1 μg/mouse, 2 times/week, *n* = 6 per group). Various immune cells were isolated from the lungs of mice by antibody conjugated magnetic beads. Each value is the mean ± SEM; *p < 0.05. CXCL17 increased the migration (**d**) and transendothelial migration (**e**) of CD11b^+^Gr-1^+^ MDSCs in vitro. GPR35 inhibitor decreased the migration (**f**) and transendothelial migration (**g**) of CD11b^+^Gr-1^+^ MDSCs induced by CXCL17. CD11b^+^Gr-1^+^ MDSCs were isolated from the lungs of normal mice (*n* = 3). PKH26-labeled CD11b^+^Gr-1^+^ MDSCs cells were seeded onto inserts (1 × 10^5^ cells in 3-μm pore insert for migration analysis). For transendothelial migration analysis, C166 cells were seeded in 3-μm pore collagen-coated inserts for confluent monolayer, and PKH26-labeled CD11b^+^Gr-1^+^ MDSCs cells (1 × 10^5^/insert) were seeded onto C166 confluent monolayer inserts, and the migration of cancer cells was assessed by fluorescence microscope. CXCL17 (1 ng/ml) were added in bottom well as chemoattractant. For blocking experiment, GPR35 inhibitor (CID2745687, 2 μM) was added in the inserts. Results are representative of at least three independent experiments, and each value is the mean ± SD of three determinations. *Significant difference between the two test groups (*p* < 0.05)

Given that soluble factor CXCL17 is mainly responsible for recruiting immune cells in the lung [22], a critical step for the formation of metastatic niche favored by the cancer [5, 23], so we speculated that CXCL17 might function through remodeling of the lung microenvironment in a paracrine manner. Intra-tracheal administration of rmCXCL17 increased the infiltration of CD11b^+^Gr-1^+^ MDSCs in the lungs of mice, but CD11b^+^Gr-1^−^ MDSCs or macrophages (CD11b^+^F4/80^+^) did not (Fig. 3a–c). CXCL17 also enhanced basal and transendothelial migration of CD11b^+^Gr-1^+^ MDSCs isolated from mice in vitro (Fig. 3d, e). The inhibitor of GPR35 (G Protein-Coupled Receptor 35), a receptor of CXCL17, prevented the stimulatory effect of CXCL17 on the enhancement of CD11b^+^Gr-1^+^ MDSCs basal and transendothelial migration (Fig. 3f, g), indicating that CXCL17 might functionally mediate the inhibition of anti-cancer immunity of the lungs in mice via a GPR35-dependent manner.

**Fig. 4:**
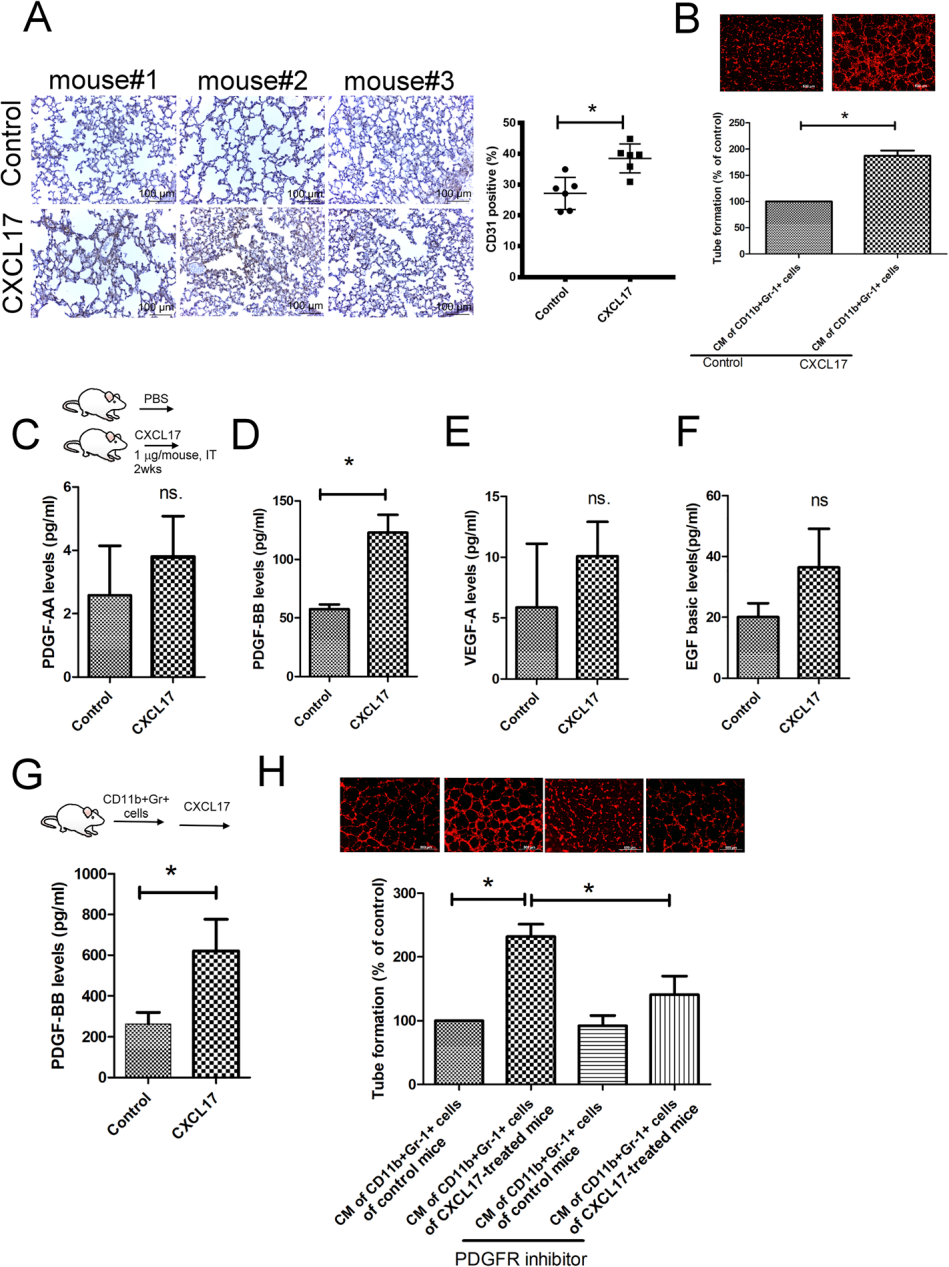
CXCL17 increases angiogenesis in lung metastatic niche by recruiting CD11b^+^Gr-1^+^ MDSCs. **a** CXCL17 increased CD31^+^ cells in the lungs of mice. Digital images of tissues were captured and analyzed with ImageJ software to calculate the percentage of positive cells (high positive + positive + low positive cells). **b** Conditioned medium (CM) (50%) of CD11b^+^Gr-1^+^ MDSCs isolated from the lungs of CXCL17-treated mice (*n* = 5) increased tube formation of C166 cells. The effect of CXCL17 in the PDGF-AA (**c**), PDGF-BB (**d**), VEGF-A (**e**), and EGF basic (**f**) secretions of CD11b^+^Gr-1^+^ MDSCs in vivo. BALB/c mice were treated with PBS or recombinant mouse CXCL17 protein by intra-tracheal administration for 14 days (1 μg/mouse, 2 times/week, *n* = 6 per group). The lungs of these mice were harvested and constrained by CD31 antibody and H&E. Alternatively, CD11b^+^Gr-1^+^ MDSCs were isolated from lungs of PBS or CXCL17 treated mice (*n* = 6 per group) by antibody conjugated magnetic beads, and CM was collected after culturing for 24 h. The expression of various angiogenic factors was assessed by Luminex Assays. **g** CXCL17 increased the expression of PDGF-BB in CD11b^+^Gr-1^+^ MDSCs isolated from the lungs of normal mice. CD11b^+^Gr-1^+^ MDSCs were isolated from the lungs of normal mice (*n* = 5) by antibody conjugated magnetic beads and treated with rmCXCL17 (10 ng/ml) for 24 h. The expression of PDGF-BB was assessed by Luminex Assays. **h** Inhibition of PDGFR-β by specific inhibitor (DMPQ-2HCl, 5 μM) prevents CD11b^+^Gr-1^+^ MDSC-mediated C166 tube formation. Results are representative of at least three independent experiments in vitro studies, and each value is the mean ± SD of three determinations. *Significant difference between the two test groups (*p* < 0.05)

Increased angiogenesis in the metastatic niche is considered a crucial event for dissemination of cancer cells invading distant organs [23, 24], and MDSCs have been implicated in orchestrating aberrant angiogenesis in metastatic niches of various cancers [25]. IHC staining of lungs of CXCL17-treated mice revealed increased CD31^+^ cells in the lungs of mice (Fig. 4a). Tube formation analysis shows that the conditioned medium (CM) of CD11b^+^Gr-1^+^ MDSCs isolated from the lungs of CXCL17-treated mice enhanced tube formation in mouse endothelial C166 cells compared to the CM of CD11b^+^Gr-1^+^ MDSCs isolated from the lungs of control mice (Fig. 4b). High-throughput screening by a Luminex system identified increased expressions of PDGF-BB expression in CD11b^+^Gr-1^+^ MDSCs isolated from lungs of CXCL17-treated mice in vivo, compared to the CD11b^+^Gr-1^+^ MDSCs isolated from the lungs of control mice. There were increased trends in the expressions of PDGF-AA, VEGF-A, and EGF basic, although they did not reach statistical significance (Fig. 4c–f). rmCXCL17 increased the expression of PDGF-BB in CD11b^+^Gr-1^+^ MDSCs isolated from lungs of normal mice in situ (Fig. 4g). Inhibitor of PDGFR-β, a specific receptor for PDGF-BB, partially decreased the stimulatory effects of CXCL17-treated CD11b^+^Gr-1^+^ MDSC’s CM in tube formation of C166 cells, revealing that MDSC-derived PDGF-BB is the mediator of angiogenesis in lung metastatic niches (Fig. 4h).

### CXCL17-derived CD11b^+^GR-1^+^ MDSC mediates the cancer-promoting effects of the lung metastatic niche, in vitro and in vivo

**Fig. 5:**
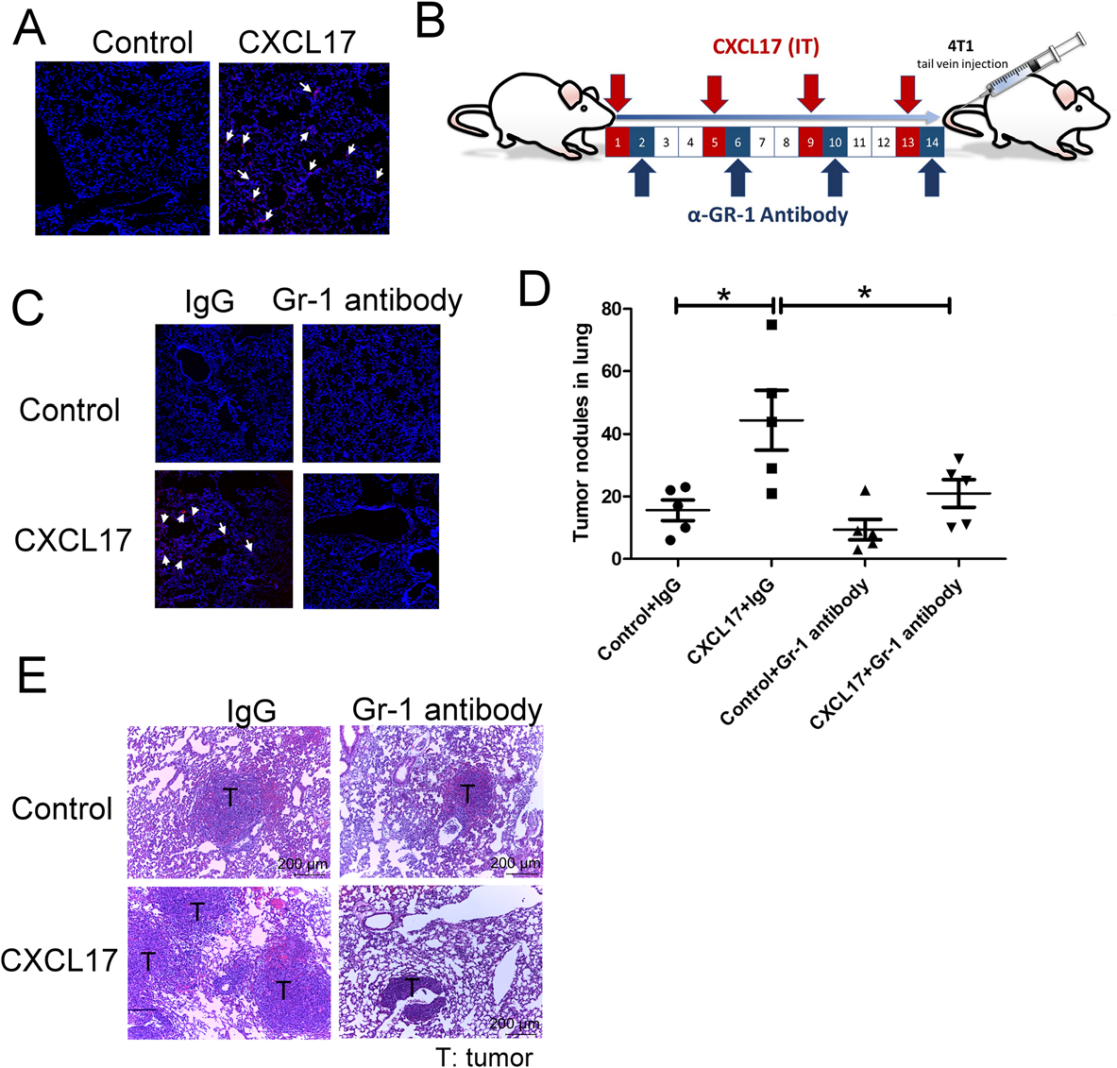
CD11b^+^Gr-1^+^ myeloid cells in lungs metastatic niche promote cancer cell colonization. **a** CXCL17 increased cancer cell extravasation into the lungs of mice. Athymic nude mice were treated with recombinant CXCL17 protein (1 μg/mouse, 2 times/week, *n* = 6 per group) for 14 days; MDA-MB-231-RFP-Luc cells were injected into mice by tail vein. The lungs of these mice were harvested after 48-h injection, then examined by a confocal microscope. **b** Scheme of CD11b^+^Gr-1^+^ depletion in the animal model. Depletion of CD11b^+^Gr-1^+^ cells decreased CXCL17-mediated cancer extravasation (**c**) and tumor nodules formation (**d**) in the lungs of mice. **e** The H&E staining of tumor sections of lungs. For MDSC depletion studies, mice (*n* = 6 per group) were treated with isotype or anti-Gr-1 antibodies (Bio X Cell) at 100 μg/mouse intraperitoneal injections every 4th day, and 4T1 cells were injected via tail vein into the mice (*n* = 6 per group). The control group received intraperitoneal injection of purified rat immunoglobulins. PKH26-labeled 4T1 cells were injected into mice by tail vein for indicated times (24 days for lung metastasis and 48 h for extravasation). The lungs of these mice were harvested and examined by a confocal microscope. Each value is the mean ± SEM; *p < 0.05. White arrows indicate cancer cells

Next, we determined if CXCL17 would also facilitate breast cancer cell extravasation and long-term lung colonization. MDA-MB-231 cells expressing RFP and luciferase were injected into the tail veins of mice after CXCL17 treatment. A significant increase of MDA-MB-231 cells was observed in the lungs of mice 48 h after implantation, showing extravasation of cancer cells into the lung (Fig. 5a). Consistently, more tumor nodules and increased size of tumors were found in the lungs of CXCL17 pre-treated mice compared with those of control mice (Fig. 2c, d). These results indicate that CXCL17 provides metastatic supporting niches for the distant spread of breast cancer cells.

Given the prominent role of CD11b^+^Gr-1^+^ MDSCs in breast cancer metastasizing to the lungs, we next examined whether depletion of CD11b^+^Gr-1^+^ MDSCs could decrease lung metastasis of breast cancer induced by CXCL17. Mouse anti-Gr-1 antibody (0.25 mg/mouse, MDSC depletion) was administrated by IP every 4 days during CXCL17 pretreatment in mice prior to PKH26-labeled 4T1 cancer cell implantation by tail vein injection (Fig. 5b). A significant decrease of 4T1 cancer cells was observed in the lungs of mice after 48-h implantation, showing that extravasation of the 4T1 cells into the lungs was reduced by MDSC depletion (Fig. 5c). Consistently, there were fewer metastatic foci and decreased tumor sizes in the lungs of anti-Gr-1 antibody treated, when compared with isotype treated, CXCL17 pre-treated mice (Fig. 5d, e).

### CD11b^+^Gr-1^+^ MDSCs facilitate cancer extravasation and survival via PDGF-BB

**Fig. 6:**
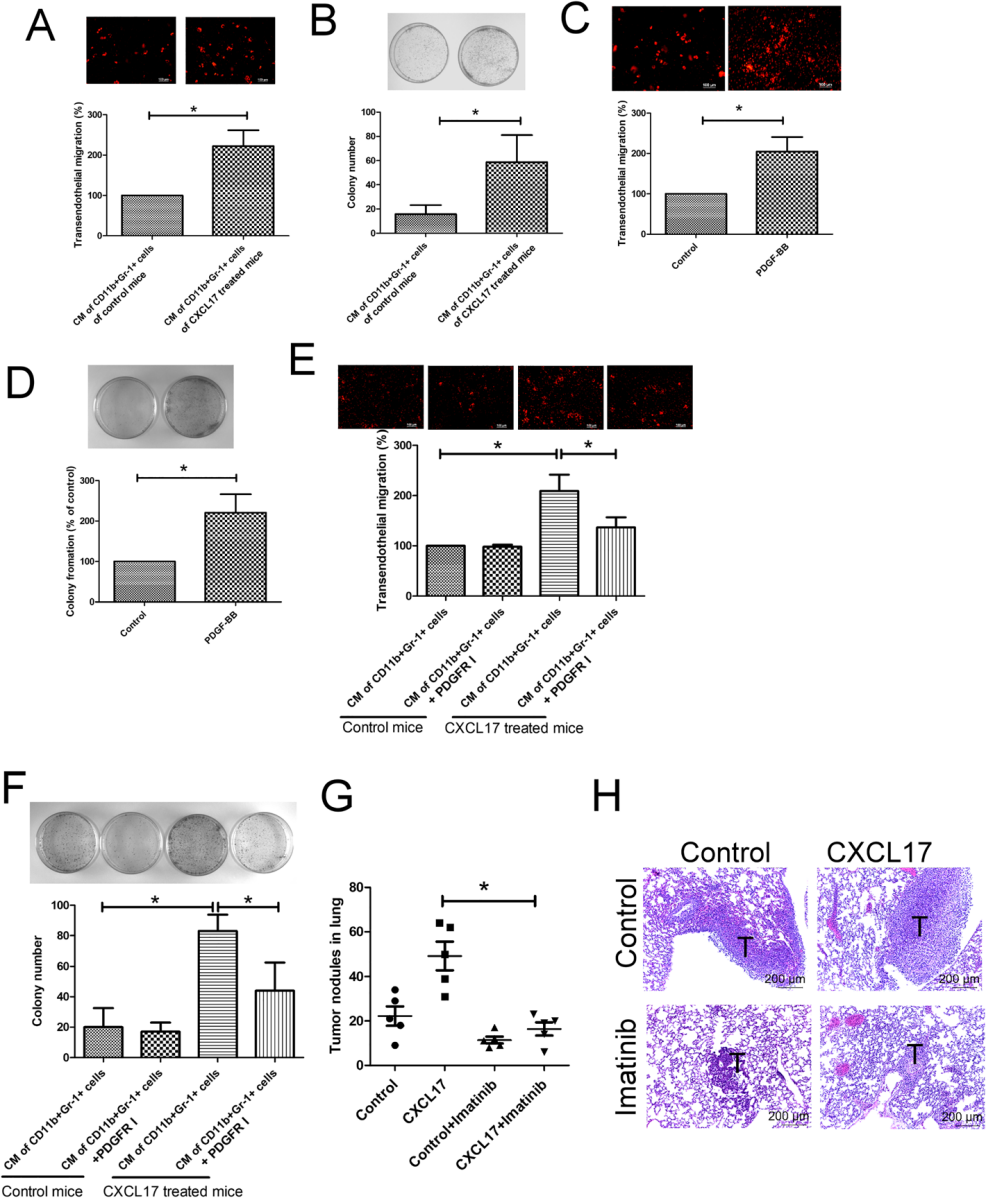
CD11b^+^Gr-1^+^ MDSCs increased breast cancer extravasation and survival via PDGF-BB. CM of CD11b^+^Gr-1^+^ MDSC isolated from the lungs of CXCL17-treated or 4T1-bearing mice (*n* = 5) increased transendothelial migration (**a**) and colony formation (**b**) of 4T1 cells. rmPDGF enhanced 4T1 cell transendothelial migration (**c**) and colony formation (**d**). Blockade of PDGFR-β prevented transendothelial migration (**e**) and colony formation (**f**) of 4T1 cells induced by CM of CD11b^+^Gr-1^+^ MDSCs. CD11b^+^Gr-1^+^ MDSC isolation and the collection of their CMs have been described in the legend of Fig. 2. C166 cells were seeded 8-μm pore collagen-coated inserts for confluent monolayer, and PKH26-labeled 4T1 cells (1 × 10^5^/insert) were seeded onto C166 confluent monolayer inserts, and the CM of CD11b^+^Gr-1^+^ MDSCs (50%) or rmPDGF-BB protein (20 ng/ml) were placed in the bottom well as chemoattractant. The migration of cancer cells was assessed by a fluorescence microscope. For colony formation analysis, 4T1 cells were treated with different CMs (50%) or PDGF-BB (20 ng/ml), and the colonies counted after staining. **g** PDGFR inhibitor imatinib decreased lung metastasis in mice (*n* = 6 per group). **h** H&E staining of lung sections. Representative lung tissue sections were stained with H&E and photographed at × 100 magnification. Results are representative of at least three independent experiments and each value is the mean ± SD of three determinations. *Significant difference between the two test groups (*p* < 0.05)

Because PDGF-BB has been implicated as being an oncogenic factor for metastasis in various cancers [26, 27], and CXCL17 provokes CD11b^+^Gr-1^+^ MDSCs to express high levels of PDGF-BB, we investigated the impact of CD11b^+^Gr-1^+^ MDSC-derived PDGF-BB in cancer extravasation, as determined by transendothelial migration analysis. We found that the CM of CD11b^+^Gr-1^+^ MDSC isolated from the lungs of CXCL17-treated mice increased the transendothelial migration of 4T1 cells (Fig. 6a). In addition, elevated colony formation of 4T1 cultured in CM of CD11b^+^Gr-1^+^ MDSCs isolated from CXCL17-treated mice further supported that CXCL17-conducted CD11b^+^Gr-1^+^ MDSC increased 4T1 cell survival (Fig. 6b). Treatment of 4T1 cells with rmPDGR-BB enhanced transendothelial migration and colony formation (Fig. 6c, d). PDGFR inhibitor prevented transendothelial migration and colony formation of 4T cells induced by CM of CD11b^+^Gr-1^+^ MDSC (Fig. 6e, f), indicating that CD11b^+^Gr-1^+^ MDSC may support breast cancer extravasation and survival by releasing PDGF-BB. Furthermore, PDGFR inhibitor imatinib decreased metastatic foci and decreased tumor sizes in the lungs of mice, when compared with isotype-treated and CXCL17 pre-treated mice (Fig. 6g, h).

## Discussion

Increasing evidence indicates that metastatic sites do not support survival of disseminated cancer cells, but are also actively and specifically primed by the primary tumor before cells arrive at the metastatic site [5, 23]. Characterization and recognition of primary cancer-derived factors that trigger vascular disruption and immune responses in the formation of lung metastatic niche in breast cancer is an important but as yet poorly defined process. In this study, we provide data that reveals the novel functions of CXCL17 on the establishment of supportive lung metastatic niches for cancer by recruiting CD11b^+^Gr-1^+^ MDSCs, which in turn support angiogenesis, cancer extravasation, and survival in new microenvironments.

Previous studies have indicated that primary cancer cells release soluble factors that either directly recruit immune cells to the metastatic niche or cooperate with niche cells to establish a metastatic environment [28, 29]. In addition, it has been reported that the primary cancer or immune cells, which would then selectively prepare the lung metastatic niche and promote cancer cells spreading to here via making the cytokine, whereas alternatively another view indicates that the cytokine is selectively made in that niche after the cancer cells have already become established [28, 30–32]. A number of studies indicate that CXCL17 can directly recruit immunosuppressive cells, such as neutrophils, macrophages, and MDSCs, to inflammatory sites and tumor tissues [18, 22, 33]. GPR35 (or CXCR8) has been reported to be the receptor of CXCL17, expressed in various immune cells, including neutrophils, T cells, monocytes, and dendritic cells, with lower expression in B cells, eosinophils, basophils, and platelets [34, 35]. In our current study, we found that CXCL17 is specifically expressed in 4T1 tumors that have metastasized to the lungs by comparing gene profiles of 4T1 tumors, which have spread to lymph glands, the intestines, and the liver. In addition, the administration of CXCL17 is favored for metastasis of breast cancer, whereas knockdown of CXCL17 prevents spontaneous spreading of breast cancer from primary site. Moreover, CXCL17 increased infiltration of CD11b^+^GR-1^+^ MDSCs into the lung metastatic niche by enhancing basal motility and transendothelial migration in a GPR35-dependent manner. This conclusion is supported by examination of the public dataset, which indicates that CXCL17 expression is negatively correlated with the distant metastasis-free survival. These results reveal that CXCL17 is involved in both lung pre-metastatic niches before cancer cell arrival and metastatic microenvironment establishment. Therefore, CXCL17 could be used as a prognostic tool as well as a therapeutic target in lung metastasis of breast cancer.

MDSCs are necessary constituents of the metastatic niches, where they play tumor-promoting, immune-suppressive, or both roles [36, 37]. Phenotypic analysis of MDSCs accumulated in the lungs of mice bearing mammary adenocarcinoma showed that CD11b^+^Gr-1^+^ MDSCs, but not CD11b^+^Gr-1^−^ MDSCs and macrophages, are recruited in metastatic lungs, indicating that CD11b^+^Gr-1^+^ MDSCs are major, specific constituents which contribute to metastatic niche formation in the lungs [12]. The granulocytic nature of CD11b^+^Gr-1^+^ MDSCs is immunosuppressive, directly inhibiting T cell function, and other myeloid and NK cells [38, 39]. Furthermore, CD11b^+^Gr-1^+^ cells increase angiogenesis in organ-specific metastatic niches to enhance tumor metastasis by BV8 expression [40, 41]. Our study confirmed that CXCL17 increased the accumulation of CD11b^+^Gr-1^+^ MDSCs, which in turn increased angiogenesis in pulmonary metastatic niches. CXCL17-induced CD11b^+^Gr-1^+^ MDSCs also supported cancer cell extravasation and survival in the lungs of mice. Moreover, depletion of CD11b^+^Gr-1^+^ MDSCs reduced angiogenesis and cancer colonization in lungs, indicating they are responsible for multiple steps of the lung metastatic cascade in breast cancer. Our work reveals a novel role of CD11b^+^Gr-1^+^ MDSCs in lung metastasis of breast cancer, without the regulatory activity on innate and acquired immune cells.

It is well established that PDGF-BB contributes to cancer progression by promoting cancer growth, migration, and tumor angiogenesis [42, 43]. PDGF-BB triggers the invasive phenotypes of cancer cells by Src signaling, including cell adhesion, migration, and invasion [26, 43]. PDGF-BB has also been shown to increase metastasis of cancer cells into the lungs and bone [44, 45]. Cancer cells, cancer-associated fibroblasts, and lymphatic and vascular endothelial cells have been implicated as being major sources of high levels of PDGF-BB in breast cancer [46]. In this study, we found that CD11b^+^Gr-1^+^ MDSCs express higher levels of PDGF-BB, which not only increases angiogenesis in metastatic lungs, but also enhances incoming metastatic tumor cells’ migration from circulation into the lungs and growth of secondary cancers therein. Furthermore, inhibition of PDGF receptor decreases lung metastasis in mice, representing the fact that PDGF-BB contributes to lung metastasis of breast cancer. Therefore, this current study verifies that PDGF-BB plays a critical role in CD11b^+^Gr-1^+^ MDSC-mediated breast cell retention in the lungs and, if targeted, may have potential therapeutic benefit to reduce lung metastasis of breast cancer.

The specific receptors that are evaluated in breast cancer in clinical practice are the estrogen receptor (ER), progesterone receptor (PR), and human epidermal growth factor 2-neu (HER2/neu) receptor [47]. These receptors are both prognostic and predictive utilities for breast cancer patients treated with targeted therapy. Therefore, when metastasis is suspected, it is important to make a biopsy not only to check recurrent disease, but also to validate specific receptor levels. The expression of CXCL17 has been indicated as being positively correlated with ER status [48]. The models including 4T1 and MDA-MB-231 breast cancer used in our study are triple-negative breast cancer; therefore, further study is required to clarify possible roles of ER, PR, or Her2/neu in the regulation of CXCL17 in breast cancer.

## Conclusions

**Fig. 7:**
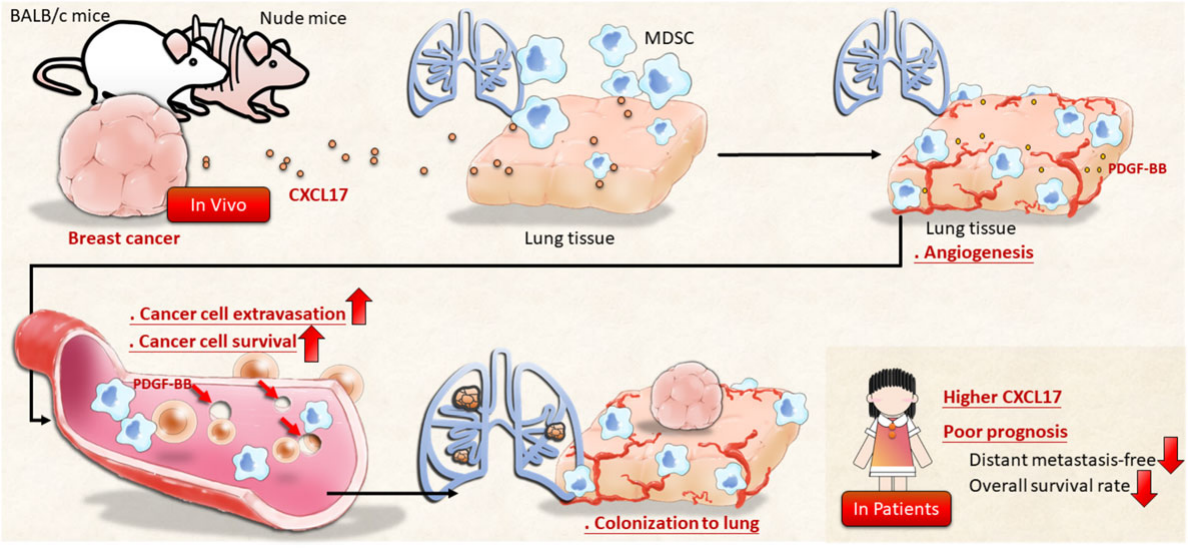
A novel mechanism underlying the contribution of primary cancer to lung metastatic niche formation in breast cancer. Primary cancer-secreted CXCL17 increases the accumulation of CD11b^+^Gr-1^+^ MDSCs in the lungs, which produce PDGF-BB, resulting in enhanced angiogenesis in the lung tissue before cancer cells’ arrival. In addition, CXCL17 also drives CD11b^+^Gr-1^+^ MDSCs to exhibit supportive activity for cancer extravasation and survival by PDGF-BB production under cancer cell arrival

Our findings identify primary breast cancer-secreted CXCL17 as an important contributor that drives the formation of the lung metastatic niche (Fig. 7). Furthermore, we discovered the presence of granulocytic CD11b^+^Gr-1^+^ myeloid cells, with pro-angiogenic activity, in the lung metastatic niche. In this setting, the potential relationship between granulocytic MDSCs to promote the extravasation and survival of cancer cells is via PDGF-BB production. Our study provides novel therapeutic options to inhibit the metastatic niche, including the targeting of CXCL17 and mechanisms promoting cancer colonization activated by CD11b^+^Gr-1^+^ MDSC-derived PDGF-BB.

## Additional file

Additional file 1: **Figure S1.** CXCL17 did not affect the cell proliferation and migration of breast cancer. The effect of CXCL17 in the cell proliferation (A), colony formation (B), and cell migration (C). Results are representative of at least three independent experiments, and each value is the mean ± SD of three determinations; ns., no significant difference with control (*p* < 0.05). (PDF 280 kb)

## Abbreviations

CXCL17: Chemokine (C-X-C motif) ligand 17
G-MDSCs: Granulocytic myeloid-derived suppressor cells
MDSCs: Myeloid-derived suppressor cells
M-MDSCs: Monocytic myeloid-derived suppressor cells
PDGF-BB: Platelet-derived growth factor-BB
