# Supplementary material for: CXCL17-derived CD11b+Gr-1+ myeloid-derived suppressor cells contribute to lung metastasis of breast cancer through platelet-derived growth factor-BB
Source: Breast Cancer Res. 2019 Feb 12;21:23. doi: 10.1186/s13058-019-1114-3 (PMC6373011; doi:10.1186/s13058-019-1114-3)
Supplement: Supplementary file 1 — Figure S1. CXCL17 did not affect the cell proliferation and migration of breast cancer. The effect of CXCL17 in the cell proliferation (A), colony formation (B), and cell migration (C). Results are representative of at least three independent experiments, and each value is the mean ± SD of three determinations; ns., no significant difference with control (p < 0.05). (PDF 280 kb) [file 13058_2019_1114_MOESM1_ESM.pdf]

Additional files

Additional file 1:

Figure S1. CXCL17 did not affect the cell proliferation and migration of breast cancer. The effect of CXCL17 in the cell proliferation (A), colony formation (B) and cell migration. Results are representative of at least three independent experiments and each value is the mean ± SD of three determinations; ns., no significant difference with control ($p<0.05$). (PDF, 200 kb).

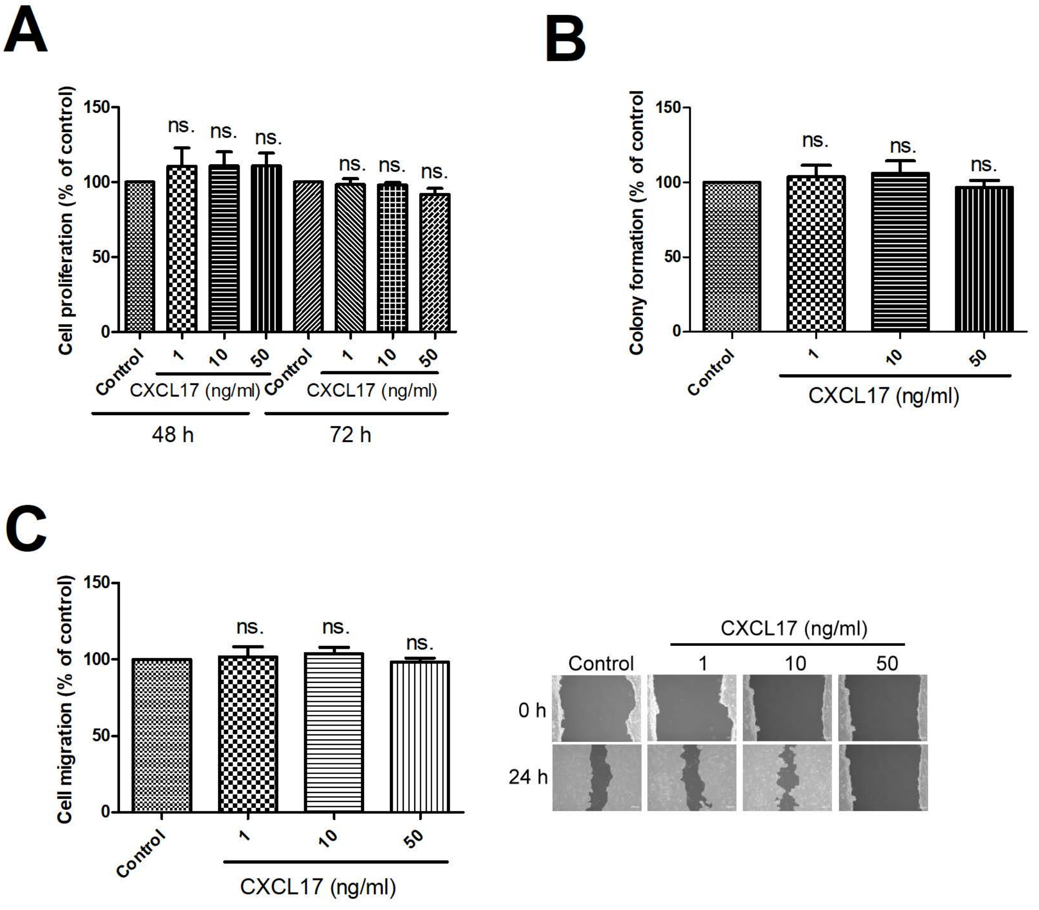
A
Cell proliferation (% of control)
150
100
50
0
ns.
Control
1
10
50
CXCL17 (ng/ml)
48 h
72 h
B
Colony formation (% of control
CXCL17 (ng/ml)
C
Cell migration (% of control)
CXCL17 (ng/ml)
Control
1
10
50
0 h
24 h

Figure S1
